# Pulsed-field ablation for refractory typical atrial flutter resistant to multiple radiofrequency ablations: A technical report

**DOI:** 10.1007/s10840-025-02183-9

**Published:** 2025-11-20

**Authors:** Shaojie Chen, Anna Neumann, Gozal Mirzayeva, Sebastian Graeger, Lukas Urbanek, David Schaack, Julian K. R. Chun, Boris Schmidt

**Affiliations:** 1 Department of Internal Medicine B (Cardiology, Angiology, Pneumology and Internal Intensive Care Medicine), University Medicine Greifswald, Greifswald, Germany; 2 Rhythmology and Clinical Cardiac Electrophysiology, Klinik Internal Medicine B (Cardiology, Angiology, Pneumology and Internal Intensive Care Medicine), University Medicine Greifswald, Ferdinand-Sauerbruch-Straße, 17475 Greifswald, Germany; 3 German Centre for Cardiovascular Research (DZHK), Partner Site Greifswald, Greifswald, Germany; 4 Cardioangiologisches Centrum Bethanien, Agaplesion Markus-Krankenhaus, Frankfurt am Main, Germany; 5 Klinik für Rhythmologie, Universitätsklinikum Schleswig-Holstein der Universität zu Lübeck, Lübeck, Germany; 6 Klinik für Kardiologie, Medizinische Klinik III, Universitätsklinikum Frankfurt, Frankfurt am Main, Germany

**Keywords:** Pulsed-Field ablation, Typical atrial flutter, Failed radiofrequency ablation, Cavotricuspid isthmus

## Abstract

This technical report presents a case with longstanding, recurrent typical atrial flutter who experienced multiple relapses despite four prior radiofrequency (RF) ablation procedures targeting the cavotricuspid isthmus (CTI). Initial RF attempts failed to achieve durable bidirectional CTI block, leading to persistent arrhythmia and significant symptom burden. Upon current admission, comprehensive electrophysiological mapping confirmed a scar-related, clockwise right atrial flutter with residual conduction across the CTI, notably on both the ventricular and inferior vena cava (IVC) sides. Given the fifth-do setting and previously scarred right atrial substrate, pulsed-field ablation (PFA) was selected as the ablation modality. Using a pentaspline PFA catheter, targeted ablation was successfully performed at the isthmus region. Residual fragmented atrial electrograms on both the ventricular and IVC sides of the isthmus were completely eliminated with paired PFA applications. High-density voltage remapping post-PFA confirmed durable electrical block across the CTI. No arrhythmia could be induced after the procedure, and the patient remained arrhythmia-free during 1-year follow-up without antiarrhythmic therapy. The procedure was complication-free, with no evidence of phrenic nerve injury, conduction system damage, or early / late coronary effects. This report suggests the integration of PFA into the therapeutic toolkit for recurrent CTI-dependent atrial flutter, particularly in patients who fail multiple conventional RF ablations. Prospective studies are warranted to validate these findings in larger populations and define long-term outcomes.

**Graphical Abstract:**

**Figure:**
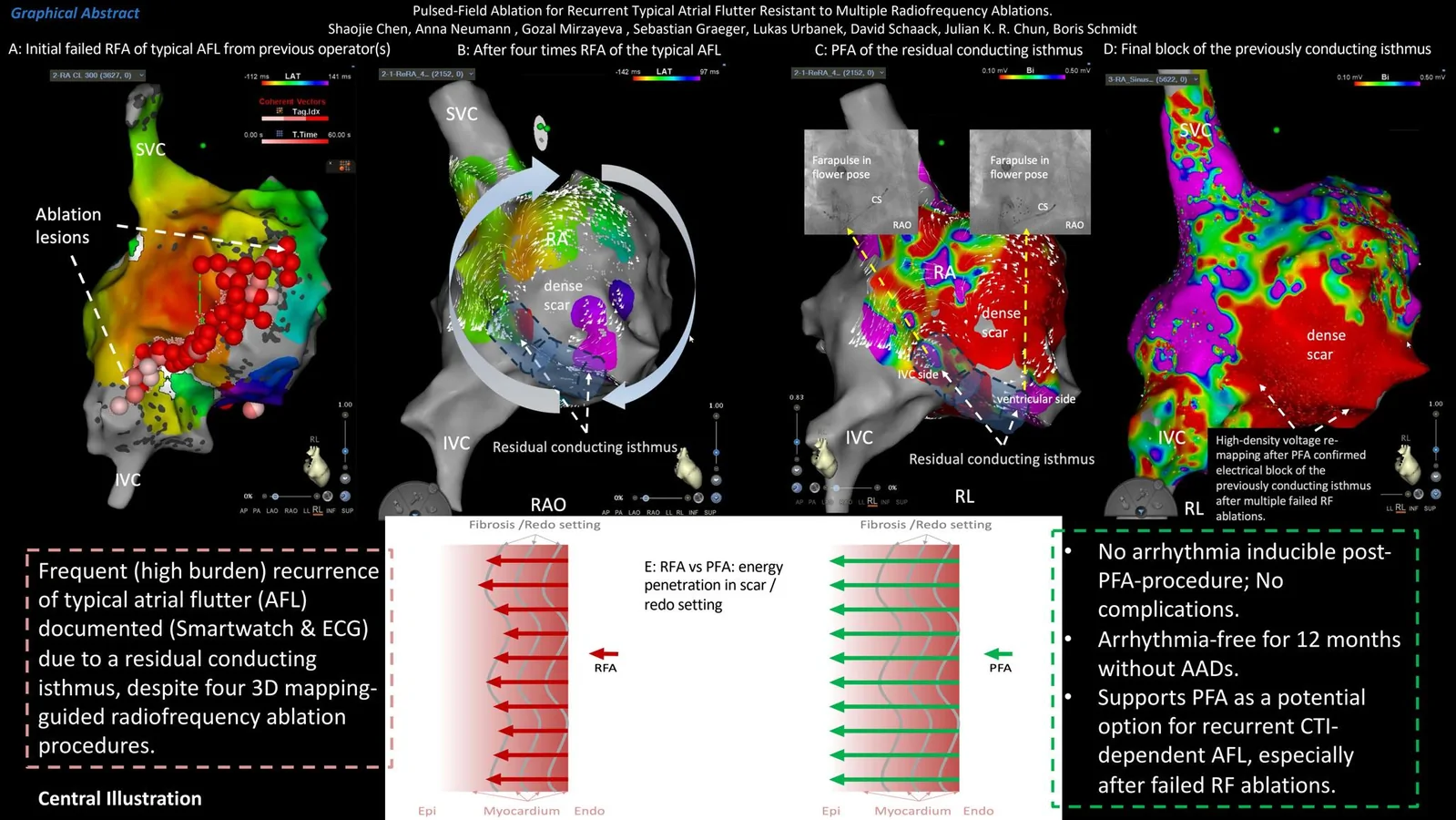
Pulsed-Field Ablation for Recurrent Typical Atrial Flutter Resistant to Multiple Radiofrequency Ablations

**Supplementary Information:**

The online version contains supplementary material available at https://doi.org/10.1007/s10840-025-02183-9.

##  Introduction

Cavotricuspid isthmus (CTI) ablation using radiofrequency (RF) energy is the standard therapy for typical atrial flutter, with high acute success rates and significant symptom relief in most patients. However, achieving durable bidirectional CTI block can be challenging in selected cases, often due to difficulties in creating contiguous and transmural lesions.

Recurrent typical atrial flutter despite multiple RF ablation procedures may indicate the need for alternative energy sources. Pulsed-field ablation (PFA), a largely nonthermal modality that preferentially ablates myocardial tissue while sparing adjacent structures, has demonstrated its efficacy for pulmonary vein isolation (PVI) in atrial fibrillation (AF) and is increasingly being used for non-PV targets [1–10]. Its unique biophysical profile may offer a therapeutic option for patients with typical atrial flutter refractory to conventional RF therapy [11].

We present a technical report of successful PFA of cavotricuspid isthmus–dependent atrial flutter in a patient with multiple prior failed RF ablations.

## Method and results

**Fig. 1:**
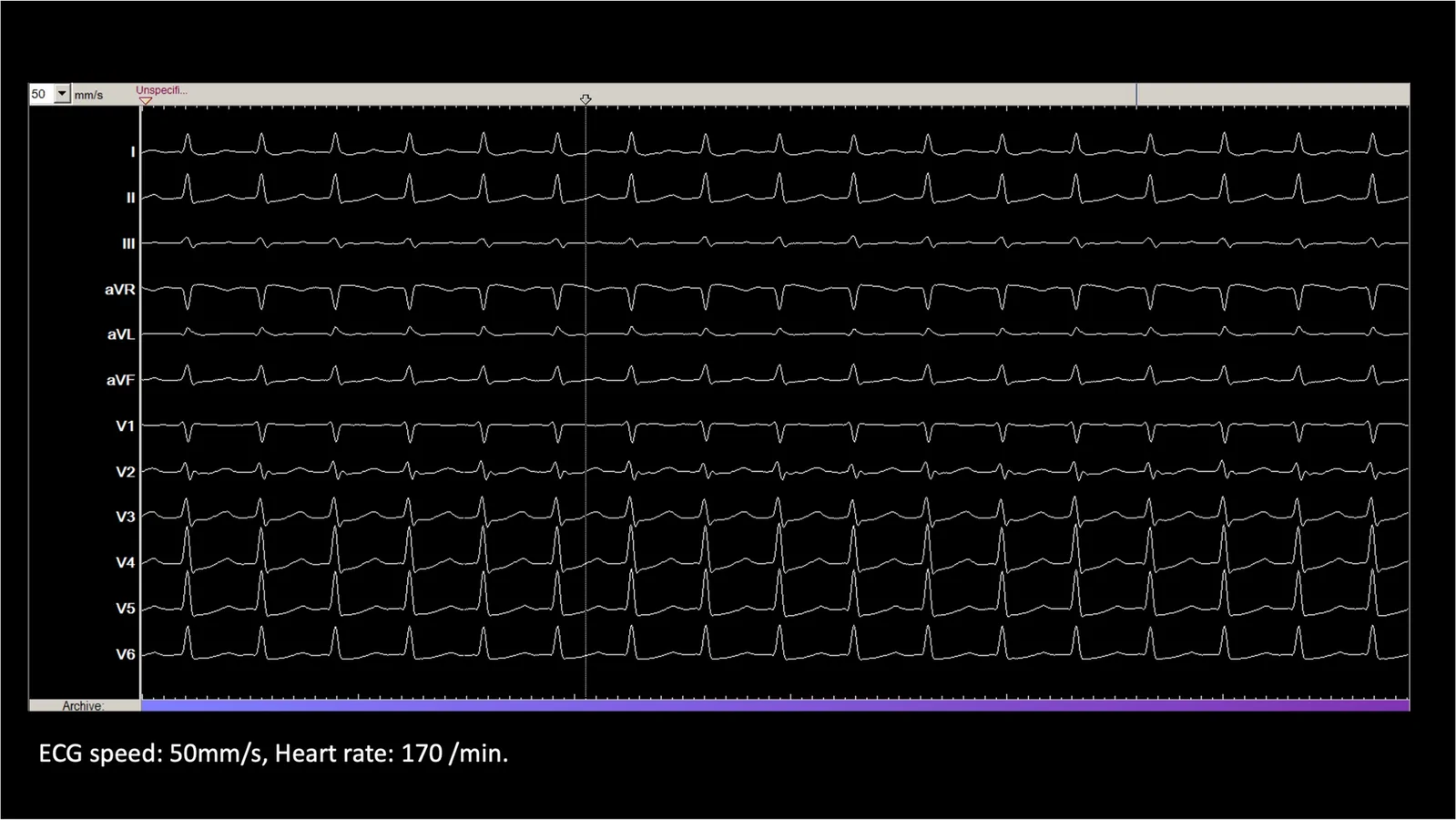
The 12-lead ECG of the recurrent tachycardia ECG speed: 50 mm/s, Heart rate: 170/min

A 45-year-old male with a history of long-standing and recurrent typical atrial flutter presented to our emergency department with a new episode of palpitations, despite having undergone four prior radiofrequency (RF) ablation procedures targeting the right atrium (RA) and cavotricuspid isthmus (CTI). A 12-lead ECG demonstrated a regular tachycardia at a rate of 170 beats per minute (Fig. 1), consistent with recordings from the patient’s smartwatch. Non-invasive maneuvers, including the Valsalva maneuver, adenosine administration, and beta-blocker therapy, failed to terminate the arrhythmia. After an informed discussion with the patient, a decision for a repeat procedure was made.

Transthoracic echocardiography showed a normal left ventricular ejection fraction (60%) with mild right atrial enlargement (RA volume index: 39 mL/m²), normal left atrial volume index (34 mL/m²), and no evidence of pulmonary hypertension. Cardiac MRI excluded infiltrative cardiomyopathies such as amyloidosis, sarcoidosis, or hemochromatosis. The recurrence of typical flutter was therefore attributed to incomplete CTI conduction block rather than underlying structural or systemic disease.

### Review of previous mapping and failed ablation by previous operator(s)

**Fig. 2:**
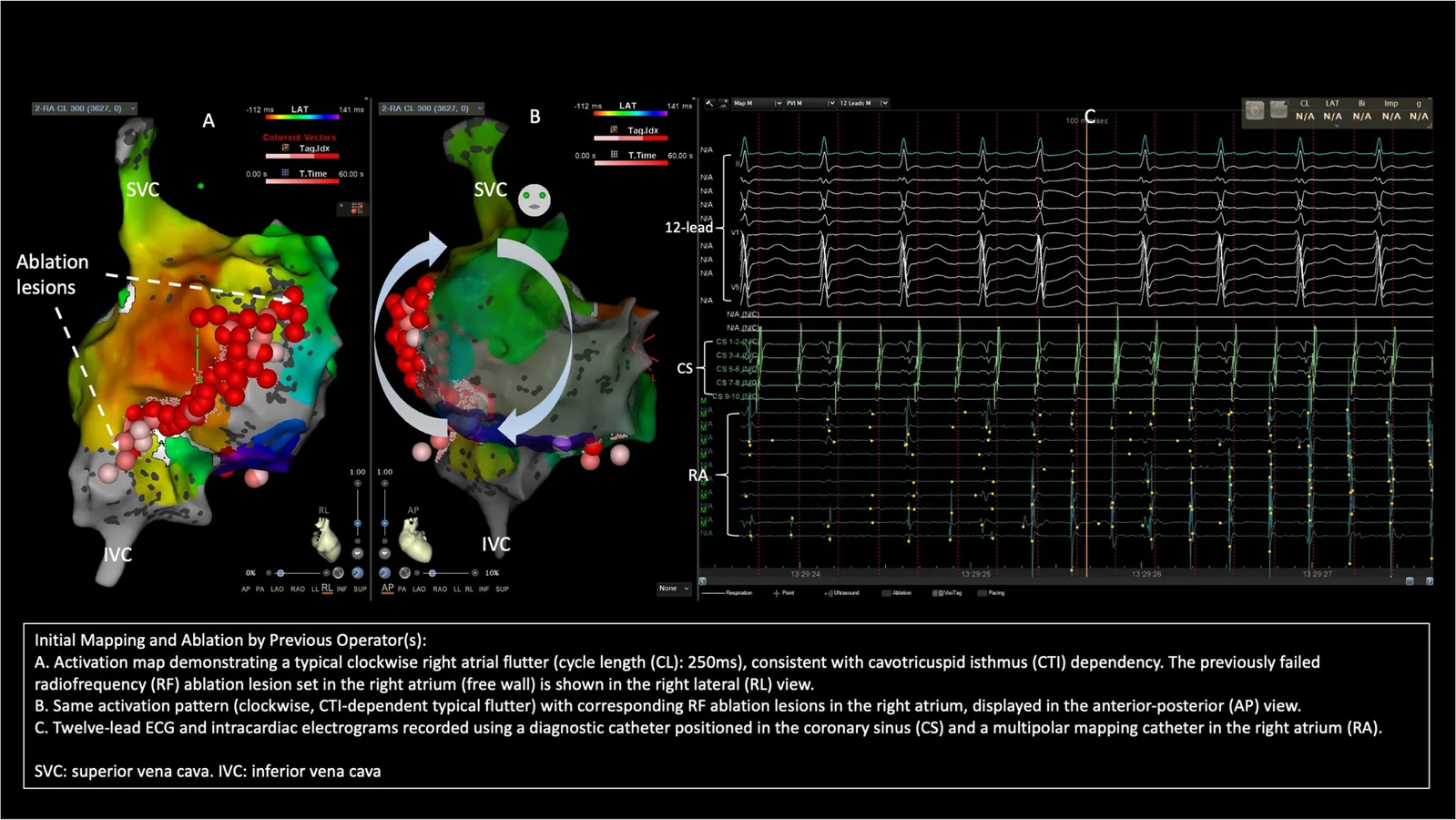
Previous procedure: Review of Initial Mapping and Ablation by Previous Operator(s) A. Activation map demonstrating a typical clockwise right atrial flutter (cycle length (CL): 250ms), consistent with cavotricuspid isthmus (CTI) dependency. The radiofrequency (RF) ablation lesion set in the right atrium (free wall) is shown in the right lateral (RL) view B. Same activation pattern (clockwise, CTI-dependent typical flutter) with corresponding RF ablation lesions in the right atrium, displayed in the anterior-posterior (AP) view C. Twelve-lead ECG and intracardiac electrograms recorded using a diagnostic catheter positioned in the coronary sinus (CS) and a multipolar mapping catheter in the right atrium (RA) SVC: superior vena cava. IVC: inferior vena cava

Figure 2 reviews the previous mapping and failed ablation procedure performed by previous operators on the patient during the clinical tachycardia episode. Figure 2A demonstrates an activation map consistent with typical clockwise right atrial flutter (cycle length (CL): 250 ms), indicating cavotricuspid isthmus (CTI) dependency. The ablation lesion set (marked by red ablation tags) in the RA (more to the free wall of the RA) is shown in the right lateral (RL) view and anterior-posterior (AP) view in Fig. 2B. Figure 2C displays the twelve-lead ECG and intracardiac electrograms recorded using a diagnostic catheter positioned in the coronary sinus (CS) and a multipolar mapping catheter in the right atrium (RA) during the typical atrial flutter. The initial radiofrequency (RF) ablation attempt was unsuccessful, and the typical atrial flutter was eventually terminated with electrical cardioversion.

### Frequent recurrence despite multiple ablation

After the initial ablation, the patient experienced early recurrence and was readmitted multiple times to different centers due to frequent arrhythmia episodes—occurring approximately 1–2 times per month. Multiple 3D-guided radiofrequency (RF) ablations were performed in the RA (at CTI and free wall of the RA according to previous medical reports); however, the arrhythmia continued to recur.

Despite four prior ablations, durable bidirectional block of the CTI was not achieved. Possible reasons include anatomical complexity of the CTI with a prominent Eustachian ridge, a local heat-sink effect from adjacent blood flow, and difficulty in maintaining stable catheter–tissue contact despite the use of a long sheath. The previously ablated tissue likely exhibited fibrosis and variable thickness, which could have limited RF lesion depth and continuity.

The patient declined antiarrhythmic drug therapy. During his current admission to our center, the patient expressed a desire for another ablation and provided informed consent.

Preclinical studies have demonstrated that PFA can create deeper and more transmural lesions in redo or scarred atrial tissue compared to RF ablation [11]. Additionally, there have been reports of uncomplicated PFA procedures performed in the RA [1]. Therefore, PFA was considered a potential treatment modality if the same arrhythmia mechanism was confirmed. These considerations were thoroughly discussed with the patient, who subsequently provided full informed consent.

### Electrophysiological evaluation during current admission

**Fig. 3:**
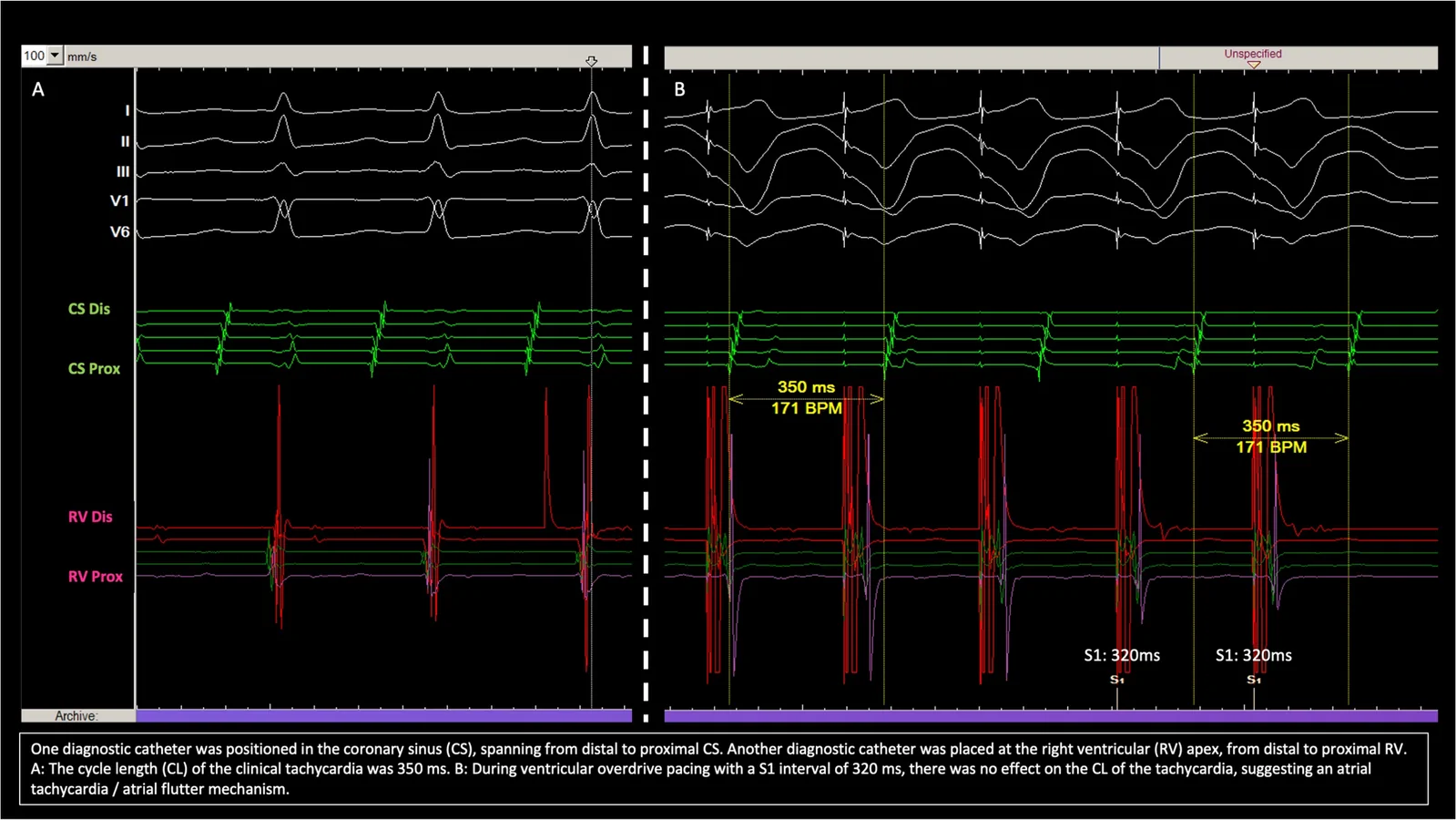
Current procedure: Ventricular overdrive pacing during tachycardia One diagnostic catheter was positioned in the coronary sinus (CS), spanning from distal to proximal CS. Another diagnostic catheter was placed at the right ventricular (RV) apex, from distal to proximal RV. A: The cycle length (CL) of the clinical tachycardia was 350 ms. B: During ventricular overdrive pacing with a S1 interval of 320 ms, there was no effect on the CL of the tachycardia, suggesting an atrial tachycardia/atrial flutter mechanism

The electrophysiological (EP) procedure was conducted by an experienced electrophysiologist (SC, primary operator) with substantial prior experience in pulsed-field ablation. the patient was under deep sedation by the administration of boluses of midazolam and continuous infusion of Propofol. EP study showed the cycle length (CL) of the clinical tachycardia was 350 ms (Fig. 3A). During ventricular overdrive pacing with a S1 interval of 320 ms, there was no effect on the CL of the tachycardia, suggesting an atrial tachycardia/atrial flutter mechanism (Fig. 3B).

**Fig. 4:**
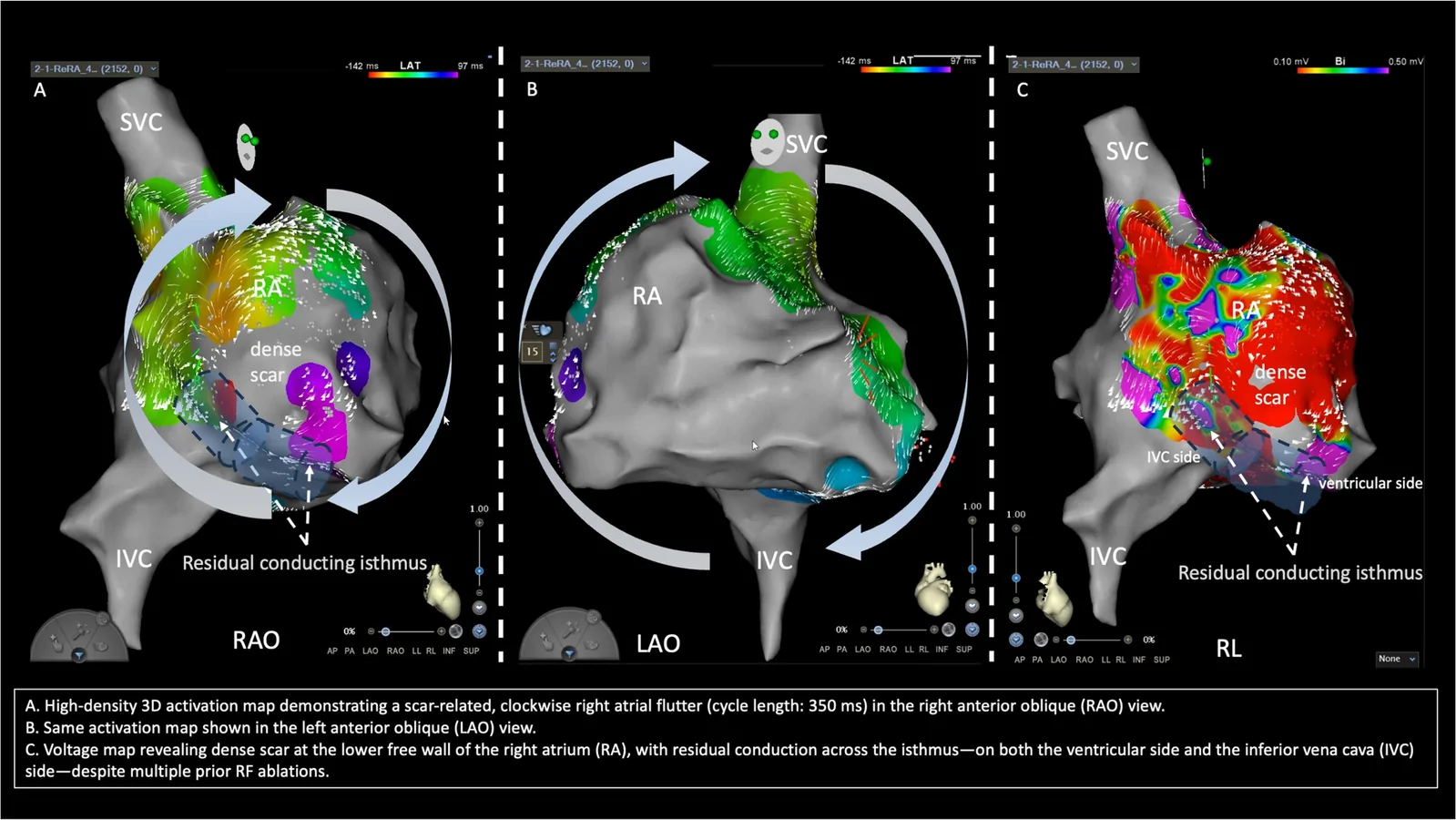
Current procedure: Electroanatomic activation and voltage maps during the tachycardia A. High-density 3D activation map demonstrating a scar-related, clockwise right atrial flutter (cycle length: 350 ms) in the right anterior oblique (RAO) view B. Same activation map shown in the left anterior oblique (LAO) view C. Voltage map revealing dense scar at the lower free wall of the right atrium (RA), with residual conduction across the isthmus—on both the ventricular side and the inferior vena cava (IVC) side—despite multiple prior RF ablations

Figure 4 shows the electroanatomic activation and voltage maps during the tachycardia. The high-density 3D activation map created with the OCTARAY™ Mapping Catheter (Carto, Biosense Webster) demonstrated a scar-related, clockwise right atrial flutter (cycle length: 350 ms) in the right anterior oblique (RAO) view and in the left anterior oblique (LAO) view (Fig. 4A-B).

The voltage map revealed dense scar at the lower free wall of the RA, with residual conduction across the isthmus—on both the ventricular side and the inferior vena cava (IVC) side—despite multiple prior RF ablations (Fig. 4C). The sites of residual conduction identified during 3D mapping were also documented under fluoroscopy in both RAO and LAO views.

### Ablation strategy and outcome

Given that this was the fifth ablation attempt in a scarred right atrium following multiple failed RF ablations for the same tachycardia mechanism (clockwise right atrial flutter), PFA was selected as the energy source for this procedure.

**Fig. 5:**
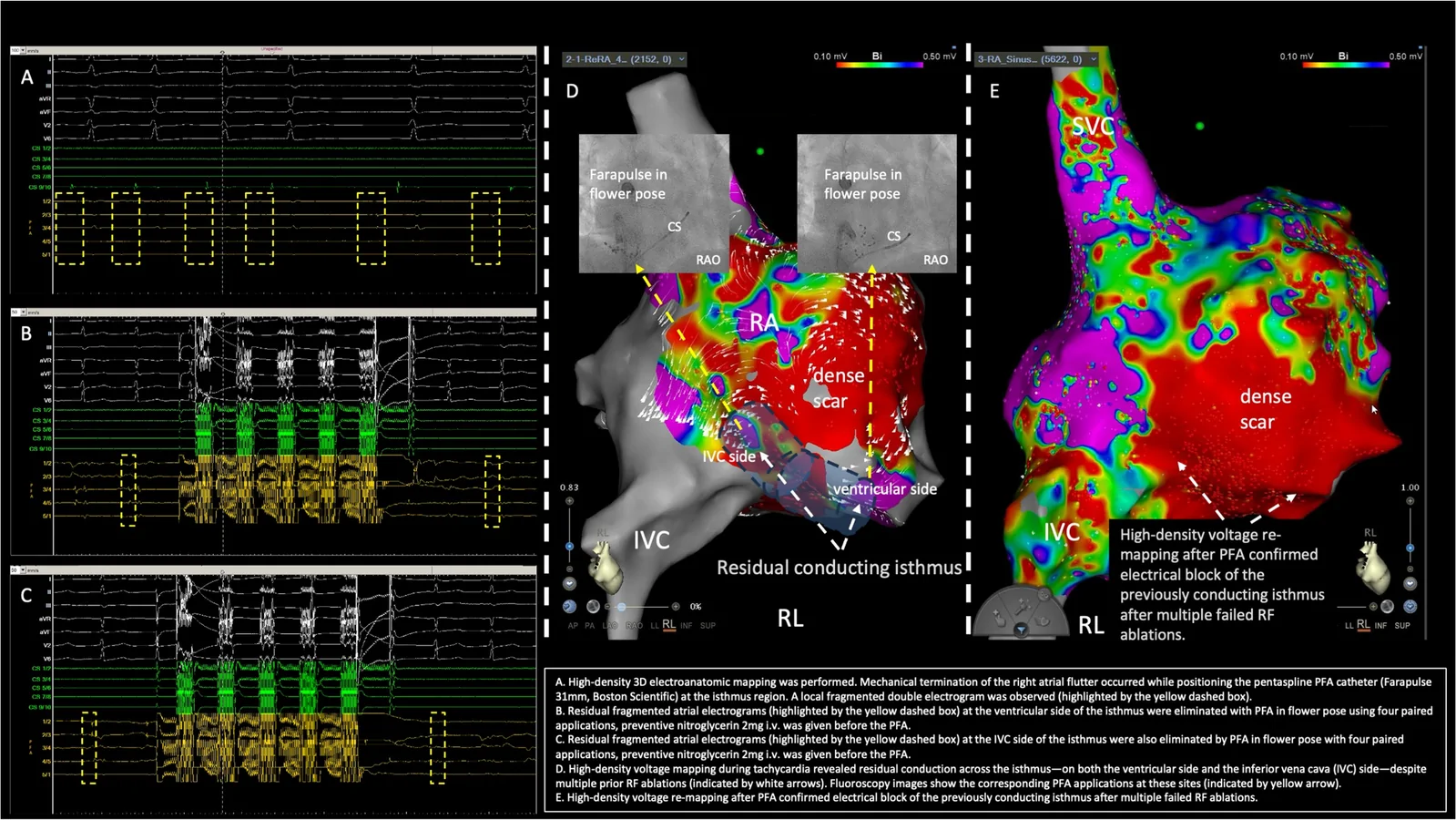
Current procedure: Pulsed-field ablation using a pentaspline catheter to eliminate residual isthmus conduction after multiple failed RF ablations A. High-density 3D electroanatomic mapping was performed. Mechanical termination of the right atrial flutter occurred while positioning the pentaspline PFA catheter (Farapulse 31 mm, Boston Scientific) at the isthmus region. Local fragmented double electrograms were observed (highlighted by the yellow dashed box) B. Residual fragmented atrial electrograms (highlighted by the yellow dashed box) at the ventricular side of the isthmus were eliminated with PFA in flower pose using four paired applications, preventive nitroglycerin 2 mg i.v. was given before the PFA C. Residual fragmented atrial electrograms (highlighted by the yellow dashed box) at the IVC side of the isthmus were also eliminated by PFA in flower pose with four paired applications, preventive nitroglycerin 2 mg i.v. was given before the PFA D. High-density voltage mapping during tachycardia revealed residual conduction across the isthmus—on both the ventricular side and the inferior vena cava (IVC) side—despite multiple prior RF ablations (indicated by white arrows). Fluoroscopy images show the corresponding PFA applications at these sites (indicated by yellow arrow) E. High-density voltage re-mapping after PFA confirmed electrical block of the previously conducting isthmus after multiple failed RF ablations

Figure 5A shows that following high-density 3D electroanatomic mapping, the right atrial flutter terminated mechanically while positioning the pentaspline PFA catheter (Farapulse 31 mm, Boston Scientific) at the cavotricuspid isthmus. Local fragmented double electrograms were observed at this site (highlighted by the yellow dashed box).

Figure 5B demonstrates that residual fragmented atrial electrograms at the ventricular side of the isthmus (yellow dashed box) were successfully eliminated using the flower configuration (Farapulse 31 mm, Boston Scientific), delivered as four paired applications. A prophylactic 2 mg intravenous dose of nitroglycerin was administered prior to energy delivery.

Figure 5C shows that residual electrograms at the IVC side of the isthmus (also highlighted by the yellow dashed box) were similarly eliminated using PFA in flower pose with four paired applications, again preceded by 2 mg of intravenous nitroglycerin.

Figure 5D presents the high-density voltage map during tachycardia, which revealed residual conduction across the isthmus—on both the ventricular and IVC sides—despite prior RF ablations (indicated by white arrows). Corresponding fluoroscopy images show the locations of the PFA applications at these residual conduction sites (yellow arrows).

Figure 5E shows the high-density voltage re-map after PFA, confirming complete electrical block of the previously conducting isthmus that had persisted despite multiple RF ablation attempts.

After ablation, a comprehensive electrophysiological (EP) study was performed. No tachycardia—including supraventricular tachycardia (SVT), atrial flutter, or atrial tachycardia—could be induced using programmed stimulation or aggressive burst pacing, both with and without isoprenaline provocation.

After PFA, intraprocedural observation revealed no evidence of phrenic nerve palsy (PNP), bradycardia, or ST elevation/depression. Complications were further excluded by echocardiography and continuous in-hospital ECG-Monitoring. The patient resumed normal daily activities, including moderate- to high-intensity training, and showed no evidence of arrhythmia recurrence—based on symptoms, or smartwatch ECG recordings, or standard ECG documentation—nor any signs of angina during the 1-year follow-up.

## Discussion

### Role of CTI Block in treating typical atrial flutter

Catheter ablation is considered the first-line therapy for typical atrial flutter, a macro-reentrant arrhythmia most commonly dependent on the CTI in the RA. The cornerstone of successful ablation in these cases is the creation of a complete and durable bidirectional conduction block across the CTI. Achieving this electrical block effectively disrupts the reentrant circuit and prevents recurrence of the arrhythmia. The procedure is typically guided by 3D electroanatomic mapping, and acute success rates with RF energy are high in first-time procedures.

### CTI block: can be challenging

While CTI ablation using RF energy is generally effective, it can become challenging in redo cases or in patients with significant atrial scarring. Fibrotic or previously ablated tissue may resist further energy penetration, resulting in incomplete lesion formation. In such settings, electrical conduction may persist across narrow surviving myocardial fibers or via protected isthmus segments, leading to continued arrhythmia recurrence. Additionally, anatomical variations, poor catheter-tissue contact, or edema from prior ablations may further compromise lesion durability.

### Potential reasons for persistent recurrence of typical atrial flutter after multiple RF ablations

Recurrent atrial flutter after multiple ablations often reflects inadequate lesion formation and/or persistent arrhythmia substrate. Incomplete electrical block of the key isthmus allows conduction to persist through viable tissue strands within or around prior ablation lines. Moreover, the heterogeneity of scar created by repeated RF lesions can lead to nonuniform anisotropic conduction, further facilitating reentry. This phenomenon underlines the importance of confirming electrical block intra-procedurally and considering alternative energy sources or strategies when conventional RF fails repeatedly.

### PFA vs. RF

PFA, a largely non-thermal energy modality based on electroporation, has shown promising results in both preclinical and early clinical studies. Unlike thermal energy sources, PFA selectively affects myocardial cells while sparing adjacent structures. In redo or heavily scarred settings where RF energy may not reliably produce transmural lesions, PFA can offer deeper and more uniform tissue penetration [11]. Given this patient’s history of multiple failed RF ablations and persistent CTI-dependent flutter, the use of PFA was a rational choice to overcome prior procedural limitations and achieve a durable block.

RF lesion formation depends on resistive and conductive heating, which can be limited in previously scarred tissue by heterogeneous impedance and heat-sink effects from adjacent vasculature. In contrast, PFA achieves irreversible electroporation by delivering ultra-short electrical pulses that create nanoscale membrane pores independent of tissue temperature or perfusion. This non-thermal mechanism enables uniform lesion depth even in fibrotic or heterogeneous substrates and may explain the complete elimination of residual CTI conduction in this case after multiple failed RF attempts.

The recent first-in-human report by Alyesh et al. described sequential, colocalized lesions created with PFA and RF using different systems (Alyesh D, Palmeri N, Sundaram S. J Interv Card Electrophysiol. 2025) [12]. In their report, the authors observed that in certain anatomically thick regions, PFA alone might not achieve full transmurality, and combining RF energy could enhance lesion depth and continuity. At first glance, this appears in contrast to the present report, in which PFA alone successfully achieved durable bidirectional conduction block across a previously refractory CTI after four failed RF attempts. However, rather than a contradiction, these findings may reflect the complementary biophysical characteristics of the two modalities. Specifically, RF efficacy may be limited in fibrotic or heat-sink–affected tissue, whereas PFA can overcome such constraints by inducing non-thermal electroporation. Conversely, in thick muscular regions where electric field density diminishes, RF may provide the additional depth needed. Taken together, these findings suggest that PFA and RF may each address the other’s limitations, supporting a tailored or sequential energy strategy in complex or redo ablation scenarios.

### The patient’s clinical outcome after the last PFA

The patient’s substantial improvement—from frequent highly symptomatic recurrences (documented by smartwatch and ECG) despite four RF procedures to sustained sinus rhythm after the last PFA session—highlights the potential clinical value of PFA in difficult atrial flutter cases. The experience of the operator may contribute to this favorable outcome; on the other hand, it also suggests that PFA achieved what prior RF ablations could not: complete isthmus block. The absence of arrhythmia recurrence over the midterm follow-up period serves as an objective indicator of procedural success and long-term efficacy. This report adds to the growing evidence that PFA may play a role in complex or redo ablation scenarios.

### Safety of PFA in right atrium

Although PFA has demonstrated a favorable safety profile, special considerations are warranted when applying it in the right atrium. First, due to the proximity of the right phrenic nerve to the CTI region, phrenic nerve capture during PFA application may be observed and transient phrenic nerve ‘’stunning’’ phenomenon may occur. Second, care should be taken to avoid energy delivery directly at the AV node or His bundle area, which could result in (transient or persistent) conduction disturbances. Third, prophylactic vasodilators such as nitroglycerin help reduce transient coronary spasm, a rare but reported adverse event during PFA. Lastly, proper catheter positioning, stable contact, and controlled number of PFA applications remain crucial to ensure effective lesion delivery and minimize possible complications.

### Limitation

This paper serves as a technical report and cannot establish the generalizable efficacy or safety of PFA for CTI ablation. The findings should be regarded as hypothesis-generating and warrant validation in larger, prospective studies.

## Summary

This technical report presents a case with recurrent typical atrial flutter who experienced multiple relapses despite four prior RF ablation procedures targeting the RA and the CTI. Previous RF attempts failed to achieve successful and durable CTI block, leading to persistent arrhythmia and significant patient’s symptom burden. Upon current admission, comprehensive electrophysiological mapping confirmed a scar-related, clockwise right atrial flutter with residual conduction across the CTI, notably on both the ventricular and inferior vena cava (IVC) sides. Given the redo setting and previously scarred right atrial substrate, PFA was selected as the ablation modality.

Using a pentaspline PFA catheter, targeted ablation was successfully performed at the isthmus region. Residual fragmented atrial electrograms on both the ventricular and IVC sides of the isthmus were completely eliminated with paired PFA applications. High-density voltage remapping post-ablation confirmed durable electrical block across the CTI. No arrhythmia could be induced after the procedure, and the patient remained arrhythmia-free during midterm follow-up without antiarrhythmic therapy. The procedure was complication-free, with no evidence of phrenic nerve injury, conduction system damage, or early/late coronary effects.

**Fig. 6:**
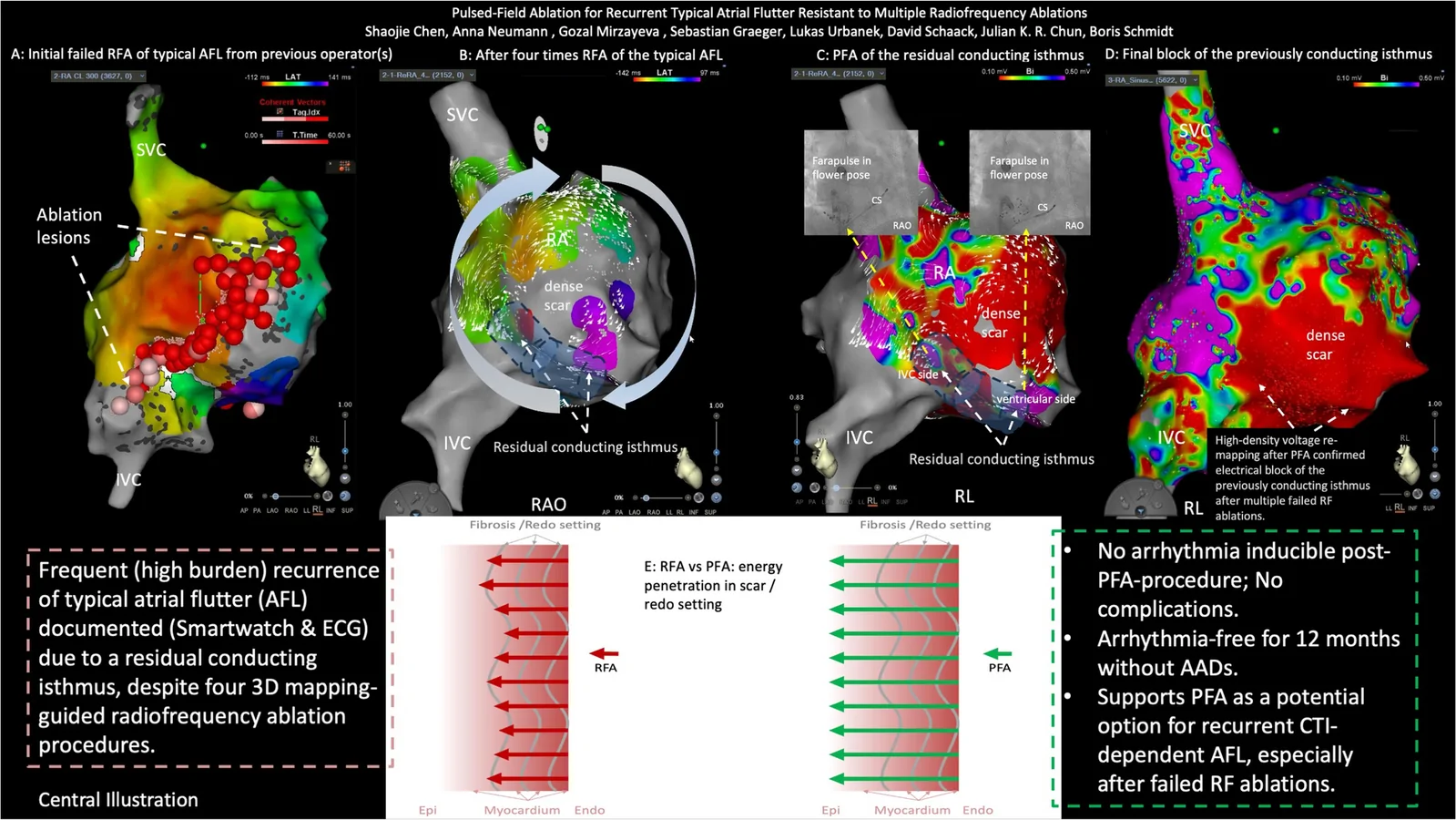
(central illustration). Pulsed-Field Ablation for Recurrent Typical Atrial Flutter Resistant to Multiple Radiofrequency Ablations **A**: Initial failed RFA (radiofrequency ablation) of typical AFL (atrial flutter) from previous operator(s) **B**: After four times RFA of the typical AFL **C**: PFA of the residual conducting isthmus **D**: Final block of the previously conducting isthmus **E**: RFA vs. PFA: energy penetration in scar/redo setting

This report suggests the integration of PFA into the therapeutic toolkit for recurrent isthmus dependent atrial flutter, particularly in patients who fail multiple conventional RF ablations (in cases where RF resistance is encountered). The take home message is illustrated in Fig. 6 (central illustration). Prospective studies are warranted to validate these findings in larger populations and define long-term outcomes.

## Supplementary Information

Below is the link to the electronic supplementary material.

Supplementary Material 1 (PDF 1.27 MB)

## Data Availability

Data are contained within the article.
