# Supplementary material for: Pulsed-field ablation for refractory typical atrial flutter resistant to multiple radiofrequency ablations: A technical report
Source: J Interv Card Electrophysiol. 2025 Nov 20;69(6):1181–9. doi: 10.1007/s10840-025-02183-9 (PMC13645872; doi:10.1007/s10840-025-02183-9)
Supplement: Supplementary file 1 [file 10840_2025_2183_MOESM1_ESM.pdf]

Pulsed-Field Ablation for Recurrent Typical Atrial Flutter Resistant to Multiple Radiofrequency Ablations.

Shaojie Chen, Anna Neumann , Gozal Mirzayeva , Sebastian Graeger, Lukas Urbanek, David Schaack, Julian K. R. Chun, Boris Schmidt

A: Initial failed RFA of typical AFL from previous operator(s)

B: After four times RFA of the typical AFL

C: PFA of the residual conducting isthmus

D: Final block of the previously conducting isthmus

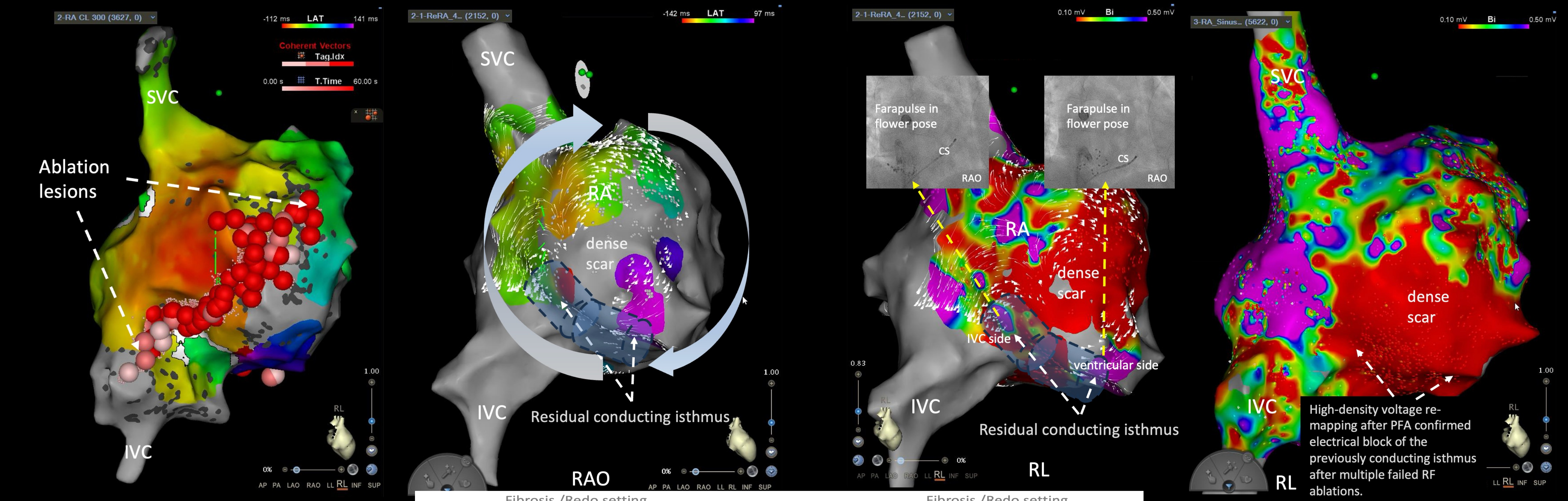

Frequent (high burden) recurrence of typical atrial flutter (AFL) documented (Smartwatch & ECG) due to a residual conducting isthmus, despite four 3D mapping-guided radiofrequency ablation procedures.

E: RFA vs PFA: energy penetration in scar / redo setting

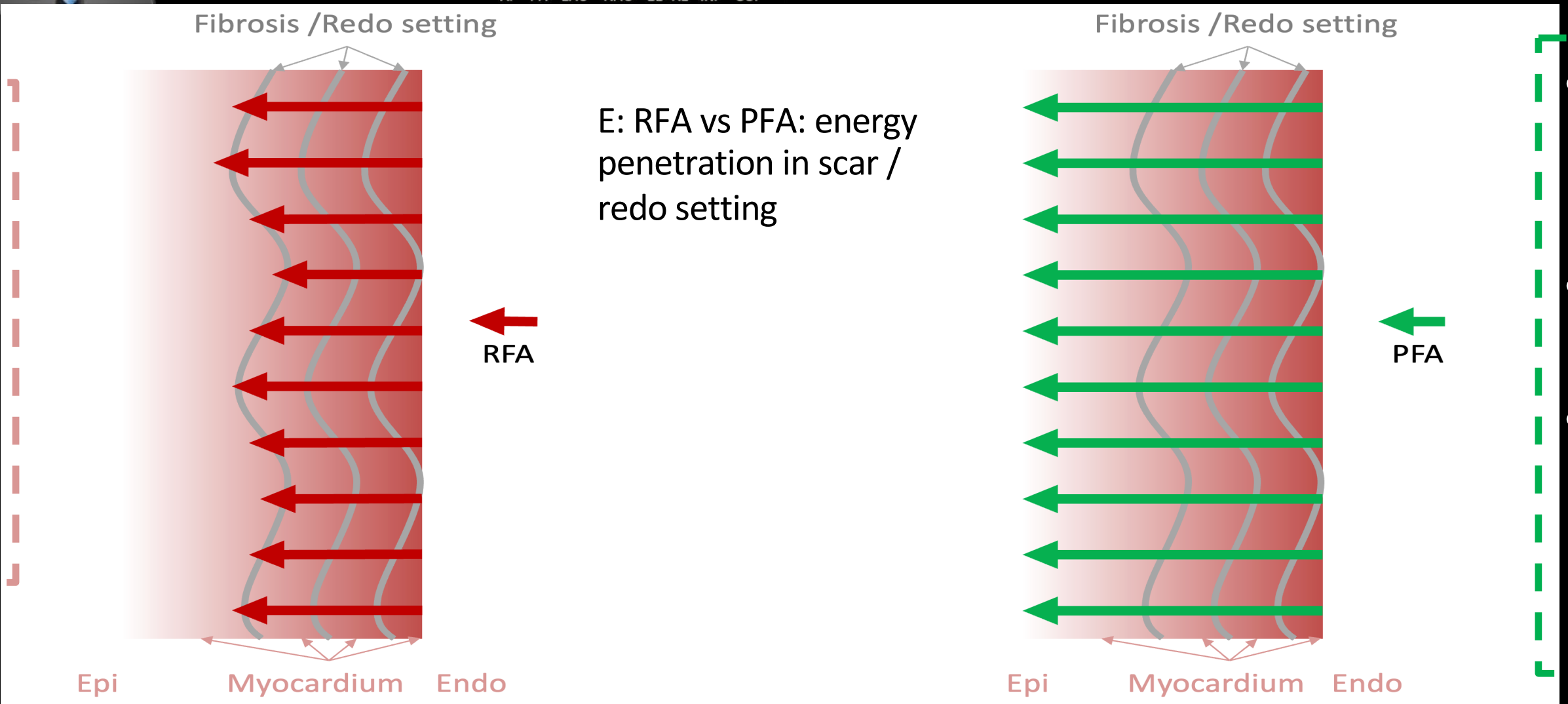

- No arrhythmia inducible post-PFA-procedure; No complications.
- Arrhythmia-free for 12 months without AADs.
- Supports PFA as a potential option for recurrent CTI-dependent AFL, especially after failed RF ablations.

Central Illustration
